# NFE2/miR-423-5p/TFF1 axis regulates high glucose-induced apoptosis in retinal pigment epithelial cells

**DOI:** 10.1186/s12860-019-0223-2

**Published:** 2019-08-27

**Authors:** Qing Xiao, Yinu Zhao, Jia Xu, Wen-Jie Li, Yu Chen, Hong-Jing Sun

**Affiliations:** 1 Department of Ophthalmology, Second Affiliated Hospital, Zhejiang University School of Medicine, No.88, Jiefang Road, Shangcheng District, Hangzhou, 310003 Zhejiang Province People’s Republic of China; 2 Department of Ophthalmology, The Third Xiangya Hospital of Central South University, Changsha, 410013 People’s Republic of China

**Keywords:** Diabetic retinopathy, miR-423-5p, TFF1, NFE2, Retinal pigment epithelial cells

## Abstract

**Background:**

A study has shown that miR-423-5p is highly expressed in proliferative diabetic retinopathy. However, the exact biological functions and mechanisms of miR-423-5p in diabetic retinopathy (DR) progression are currently unclear. This study aimed to investigate the role of miR-423-5p in DR and the underlying mechanism.

**Results:**

Our data demonstrate that the expression of miR-423-5p is significantly increased in HG-induced RPE cells and DR patient plasma. Moreover, the overexpression of miR-423-5p exacerbates HG-induced apoptosis. Mechanistically, our results provide evidence that miR-423-5p directly targets TFF1. MiR-423-5p exerts its effect on HG-induced apoptosis in RPE cells through TFF1, and the NF-κB pathway is involved in the regulatory mechanism. Further analysis revealed that the transcription factor NFE2 regulates miR-423-5p promoter activity. In addition, NFE2 regulates the levels of TFF1 and NF-κB pathway-associated proteins by regulating the expression of miR-423-5p.

**Conclusion:**

The NFE2-miR-423-5p-TFF1 axis is a novel molecular mechanism and provides a new direction for the study and treatment of DR.

## Background

Diabetic retinopathy (DR) is a common complication of diabetes. With the continuous increase in the incidence of diabetes in recent years, the number of DR patients has also increased dramatically [1], and now accounts for approximately 35% of the total number of diabetic patients worldwide. As a fundus lesion with specific changes, it is generally believed that hyperglycaemia is a key risk factor for the development of DR [2]. The treatment mainly involves controlling blood sugar and surgical methods. However, this treatment method cannot completely prevent the pathological process of retinal micro vessels in DR and seriously affects the prognosis and quality of life of the patient [3]. Therefore, in order to achieve early prevention and treatment of DR, finding effective indicators and targets has become an urgent task in current DR research.

MicroRNAs (miRNAs) is a type of endogenous, highly conserved, small molecule non-coding RNA that widely expressed in a variety of human cells; miRNAs are involved in the regulation of biological events such as cell proliferation, differentiation, migration, and apoptosis [4, 5]. The regulatory role and application value of miRNAs in various human diseases have gradually been revealed. In recent decades, many abnormally expressed miRNAs have been identified in DR [6, 7]. Currently, numerous studies have found that many miRNAs are specifically expressed in the retina tissue, which is closely related to the growth, development, structure and function of the retina [6, 7]. For instance, miR-34c and miR-34b-3p are involved in the pathogenesis of DR by activating the p53 signalling pathway [8]. Moreover, miR-423-5p is highly expressed in proliferative diabetic retinopathy (PDR) [9]. However, the exact biological functions and mechanisms of miR-423-5p in DR progression are currently unclear.

In present study, we provided evidences that NFE2 and miR-423-5p were upregulated in DR patients, while TFF1 was downregulated. Furthermore, this study established an HG-induced model in ARPE-19 and RPE-J cells to investigate the function of miR-423-5p in HG-induced apoptosis. In terms of mechanisms, we investigated miR-423-5p and its target TFF1. Furthermore, the upstream regulatory relationship of the transcription factor NFE2 and miR-423-5p was investigated. These findings reveal novel mechanisms and provide potential treatment strategies for DR.

## Results

### MiR-423-5p is upregulated in DR and exacerbates high glucose-induced apoptosis in RPE cells

**Fig. 1:**
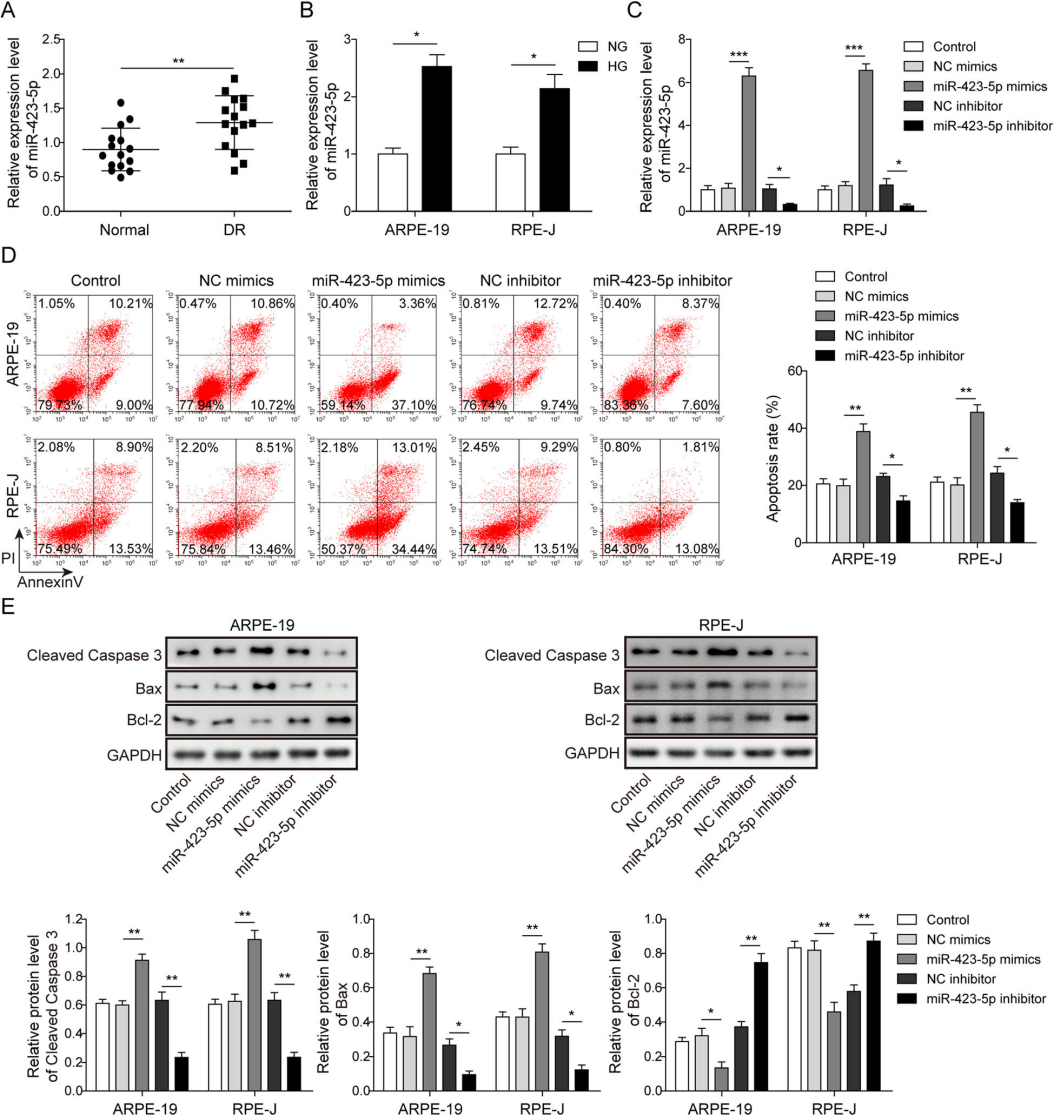
MiR-423-5p is upregulated in DR and exacerbates high glucose-induced apoptosis in RPE cells. **a** The relative levels of miR-423-5p were measured by qRT-PCR in the normal group and DR group showed that miR-423-5p was upregulated in DR group. **b** The relative levels of miR-423-5p were measured by qRT-PCR in ARPE-19 and RPE-J cells treated with NG or HG. The data demonstrated that miR-423-5p was upregulated in HG-treated cells. For **c**-**e**, ARPE-19 and RPE-J cells were transfected with NC mimics, miR-423-5p mimics, NC inhibitor or miR-423-5p inhibitor. **c** qRT-PCR analysis of miR-423-5p in ARPE-19 and RPE-J cells after transfection with miR-423-5p mimics, NC mimics, miR-185-3p inhibitor, or NC inhibitor. Transfection with miR-423-5p mimics successfully boosted the expression of miR-423-5p, while transfection with miR-423-5p inhibitor reduced the expression of miR-423-5p. **d** Apoptosis rates of ARPE-19 and RPE-J cells were measured by Annexin V/PI staining and flow cytometry. The results suggested that overexpression of miR-423-5p resulted in a significant increase in HG-induced apoptosis, while HG-induced apoptosis was significantly repressed by silencing miR-423-5p. **e** The relative protein levels of cleaved-caspase 3, Bax and Bcl-2 were assessed by Western blotting, which indicated that transfection of the miR-423-5p mimic significantly upregulated the protein levels of cleaved-caspase 3 and Bax, and downregulated the level of Bcl-2. However, transfection of a miR-423-5p inhibitor led to opposite effect. Data are presented as mean ± SD. Three replicate wells were used per sample in each experiment and experiments were replicated three times. * *P* < 0.05; ** *P* < 0.01; *** *P* < 0.001

We used qRT-PCR to detect the expression of miR-423-5p in 30 collected plasma samples (including 15 DR patients and 15 normal controls) and found that miR-423-5p was significantly upregulated in the plasma of DR patients (Fig. 1a). ARPE-19 and RPE-J cells were cultured in 5 mM (NG group) and 35 mM (HG group) D-glucose for 48 h, respectively. The expression of miR-423-5p was measured by qRT-PCR. As shown in Fig. 1b, the expression levels of miR-423-5p were significantly increased in the HG group. To explore the role of miR-423-5p in HG-induced apoptosis of RPE cells, an NC mimic, miR-423-5p mimic, NC inhibitor or miR-423-5p inhibitor was transfected into ARPE-19 and RPE-J cells. The results of the qRT-PCR showed that transfection of the miR-423-5p mimic significantly increased the expression of miR-423-5p, while transfection of the miR-423-5p inhibitor decreased the expression of miR-423-5p (Fig. 1c). The flow cytometry data suggested that overexpression of miR-423-5p resulted in a significant increase in HG-induced apoptosis in ARPE-19 and RPE-J cells, while HG-induced apoptosis was significantly repressed by silencing miR-423-5p (*P*<0.05, Fig. 1d). Moreover, the results of the Western blotting assay indicated that transfection of the miR-423-5p mimic significantly upregulated the protein levels of cleaved-caspase 3 and Bax (*P*<0.05), and downregulated the level of Bcl-2 (*P*<0.05, Fig. 1e). However, silencing miR-423-5p by transfection of a miR-423-5p inhibitor resulted in the opposite effect (*P*<0.05, Fig. 1e). Taken together, these results show that the level of miR-423-5p is increased in HG-cultured ARPE-19 and RPE-J cells, and promotes apoptosis.

### TFF1 targets miR-423-5p in ARPE-19 cells

**Fig. 2:**
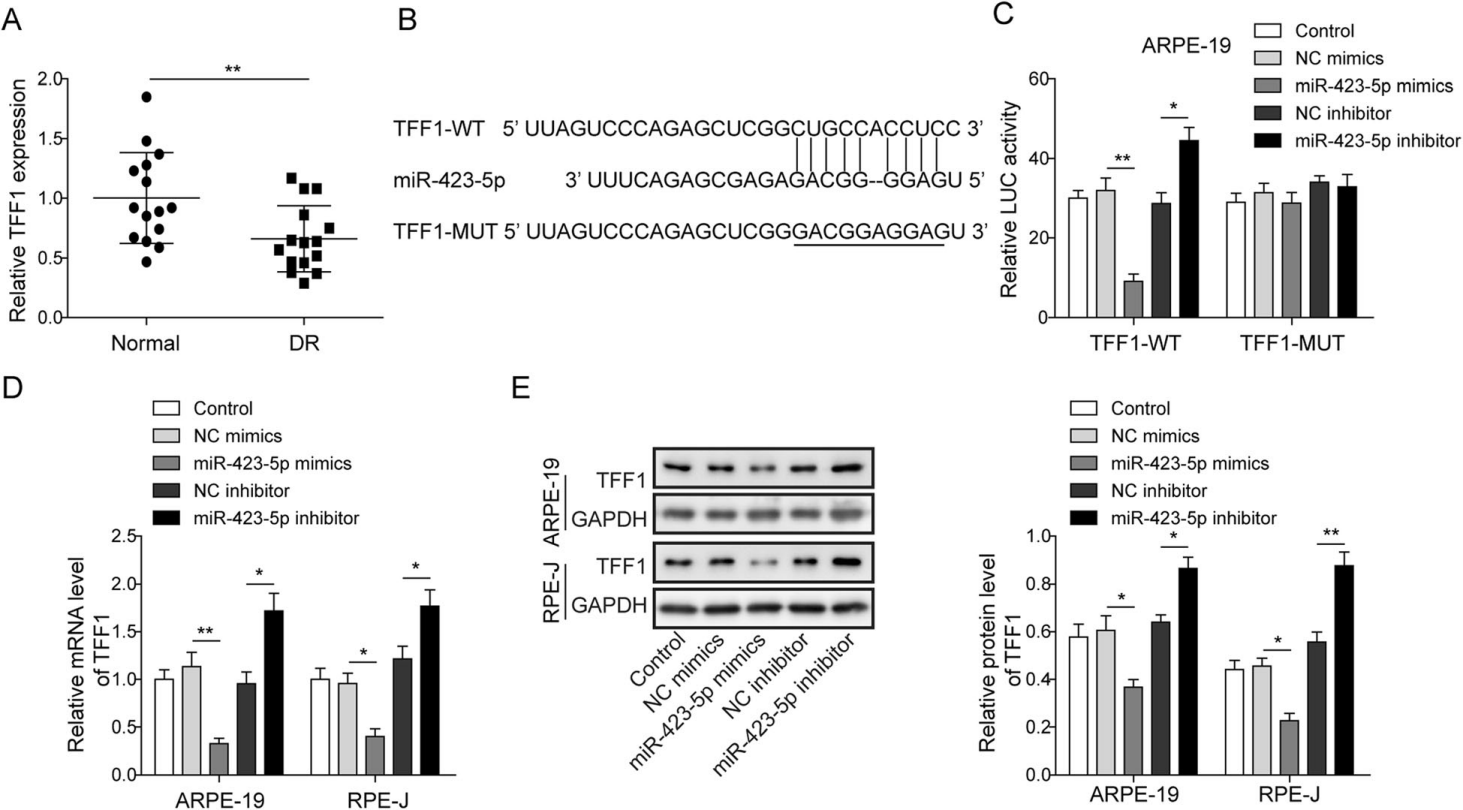
TFF1 targets miR-423-5p in ARPE-19 cells. **a** The relative levels of TFF1 were measured by qRT-PCR in the normal group and DR group showed that miR-423-5p was downregulated in DR group. **b** The predicted binding sites of miR-423-5p in the TFF1 3′-UTR. **c** The luciferase reporter vector containing TFF1-WT or TFF1-MUT was co-transfected with NC mimics, miR-423-5p mimics, NC inhibitor or miR-423-5p inhibitor. The relative luciferase activity is the ratio of the luciferase activity in each test cell sample to that in the control cell sample. The signal intensity of luciferase was lowest in TFF1-WT cells transfected with miR-185-3p mimics, and the miR-423-5p inhibitor significantly enhanced the luciferase activity. For **d** and **e**, ARPE-19 and RPE-J cells were transfected with NC mimics, miR-423-5p mimics, NC inhibitor or miR-423-5p inhibitor. The relative mRNA (**d**) and protein (**e**) levels of TFF1 were measured by qRT-PCR and Western blot, respectively. The data revealed that transfection of the miR-423-5p mimics significantly reduced expression of TFF1, while transfection of the miR-423-5p inhibitor increased the TFF1 expression. Data are presented as mean ± SD. Three replicate wells were used per sample in each experiment and experiments were replicated three times. * *P* < 0.05; ** *P* < 0.01

To further explore the underlying mechanism by which miR-423-5p regulates HG-mediated apoptosis. Using qRT-PCR to detect TFF1 expression in plasma samples from DR patients and controls, we found that TFF1 expression was significantly downregulated in patients with DR compared with healthy controls (*P* < 0.01, Fig. 2a). A bioinformatic analysis was performed to predict the binding sites of TFF1 targeted by miR-423-5p, which is located in the 3′-UTR (Fig. 2b). A luciferase reporter assay indicated that luciferase activity was significantly upregulated when ARPE-19 cells were co-transfected with TFF1-WT and the miR-423-5p mimic, while co-transfection of TFF1-MUT and the miR-423-5p inhibitor significantly enhanced the luciferase activity (*P*<0.05, Fig. 2c). However, the luciferase activity was not significantly different when the ARPE-19 cells were co-transfected with MUT and the miR-423-5p mimic or the miR-423-5p inhibitor (Fig. 2d). Subsequently, qRT-PCR and Western blotting were used to measure the effects of miR-423-5p-induced dysregulation of TFF1 expression. The qRT-PCR data indicated that transfection of the miR-423-5p mimic significantly reduced the relative mRNA levels of TFF1, while transfection of the miR-423-5p inhibitor increased the TFF1 levels in both ARPE-19 and RPE-J cells (*P*<0.05, Fig. 2d). Furthermore, the protein level trend was consistent with the trend of the mRNA levels (*P*<0.05, Fig. 2e). These results were consistent with the trend of qRT-PCR. Collectively, the above results show that miR-423-5p directly targets TFF1 and negatively regulates TFF1 expression.

### Overexpression of TFF1 restores miR-423-5p-mediated HG-induced apoptosis in RPE cells

**Fig. 3:**
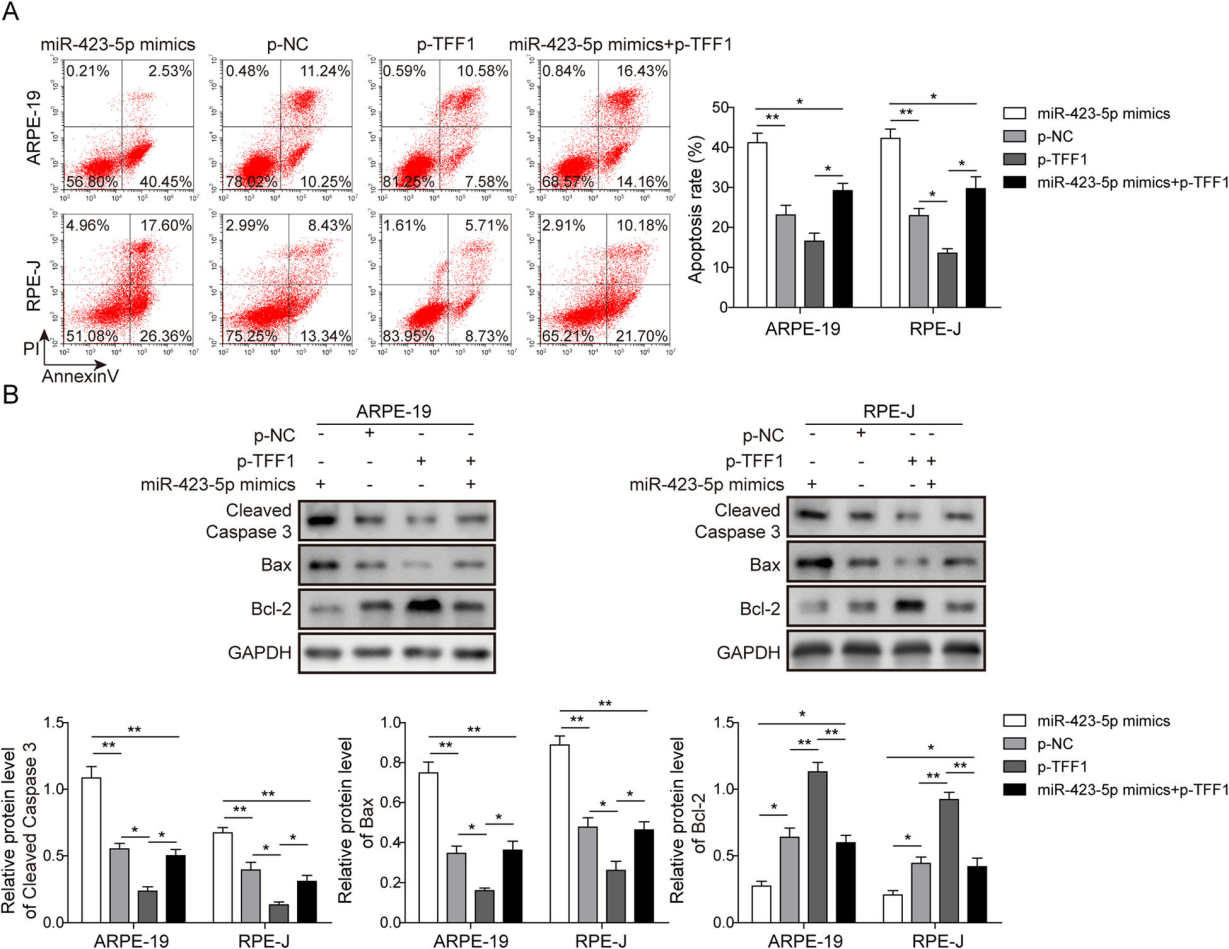
Overexpression of TFF1 restores miR-423-5p-mediated high glucose-induced apoptosis in RPE cells. ARPE-19 and RPE-J cells were divided into four groups: miR-423-5p mimic group, p-NC group, p-TFF1 group and miR-423-5p mimic group + p-TFF1 group. **a** The apoptosis rate was assessed by Annexin V/PI staining and flow cytometry. The results showed that overexpression of TFF1 significantly inhibited HG-induced apoptosis, and the overexpression of TFF1 partially restored the enhancing effects of miR-423-5p on apoptosis. **b** The relative protein levels of cleaved-caspase 3, Bax and Bcl-2 were measured by Western blot. The results showed that the transfection of p-TFF1 significantly downregulated the levels of caspase 3 and Bax and upregulated Bcl-2 levels. However, co-transfection of the miR-423-5p mimic and p-TFF1 reduced the effect of p-TFF1. Data are presented as mean ± SD. Three replicate wells were used per sample in each experiment and experiments were replicated three times. * *P* < 0.05; ** *P* < 0.01

In this study, miR-423-5p mimic, TFF1 overexpression (p-TFF1) and control plasmids (p-NC) were transfected into ARPE-19 and RPE-J cells; in addition, the miR-423-5p mimic and p-TFF1 were co-transfected into ARPE-19 and RPE-J cells. As expected, the overexpression of TFF1 significantly inhibited HG-induced apoptosis, and the overexpression of TFF1 partially restored the enhancing effects of miR-423-5p on apoptosis (*P*<0.05, Fig. 3a). Moreover, a Western blotting assay was conducted to measure the protein levels of caspase 3, Bax and Bcl-2. The results showed that the transfection of p-TFF1 significantly downregulated the levels of caspase 3 and Bax, and upregulated Bcl-2 levels (*P*<0.05, Fig. 3b). However, co-transfection of the miR-423-5p mimic and p-TFF1 reduced the regulation of p-TFF1 (*P*<0.05, Fig. 3b). Taken together, these results indicate that the overexpression of TFF1 alleviates miR-423-5p-mediated HG-induced apoptosis in RPE cells.

### Knockdown of TFF1 activates the NF-κB pathway and promoted HG-induced apoptosis in RPE cells

**Fig. 4:**
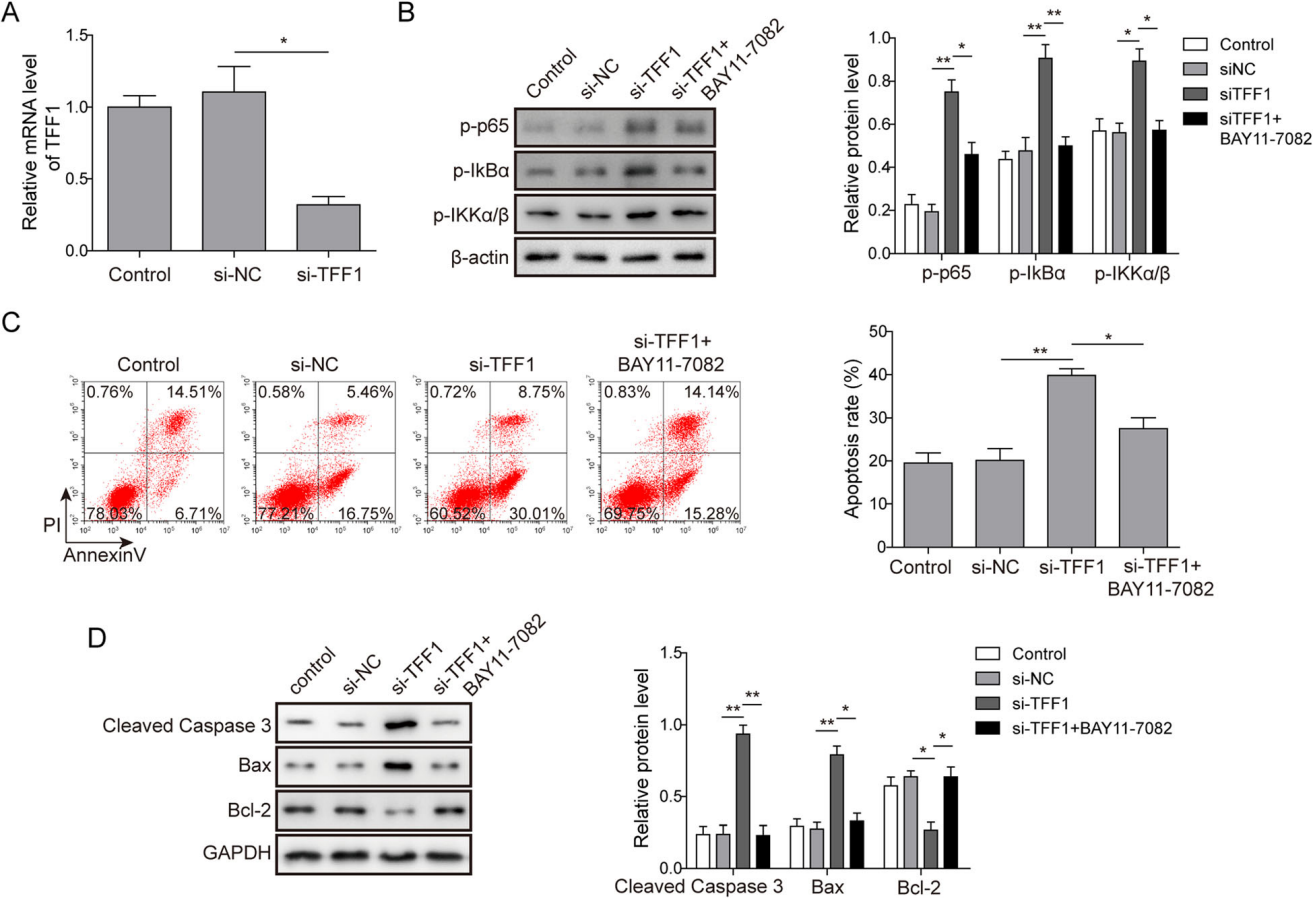
Knockdown of TFF1 activates NF-κB pathway and promotes high glucose-induced apoptosis in RPE cells. **a** The relative mRNA levels of TFF1 were measured by qRT-PCR transfection with si-TFF1 reduced the expression of TFF1 in ARPE-19 cells. For (**b**-**d**), ARPE-19 cells were divided into four groups: control group, si-NC group, si-TFF1 group and si-TFF1 + BAY11–7082 group. **b** The relative protein levels of p-p65, p-IκBα and p-IKKα/β were assessed by Western blot, which revealed that si-TFF1 promoted the phosphorylation of NF-κB pathway-associated proteins. **c** The apoptosis rate was assessed by Annexin V/PI staining and flow cytometry. The data indicated that knockdown of TFF1 significantly promoted HG-induced apoptosis, while BAY 11–7082 (inhibitor of NF-κB pathway) restored the apoptosis rate induced by si-TFF1. **d** The relative protein levels of cleaved-caspase 3, Bax and Bcl-2 were assessed by Western blot, whose data suggested that si-TFF1 significantly increased the expression of cleaved-caspase 3 and Bax and inhibited Bcl-2 expression, while BAY 11–7082 treatment reversed this effect. Data are presented as mean ± SD. Three replicate wells were used per sample in each experiment and experiments were replicated three times. * *P* < 0.05; ** *P* < 0.01

To investigate whether NF-κB is involved in the regulation of the miR-423-5p/TFF1 axis in HG-induced apoptosis in RPE cells, we established stably transfected ARPE-19 cells with si-TFF1 (*P*<0.05, Fig. 4a). Subsequently, the levels of NF-κB pathway-related proteins p-p65, p-IκBα, and p-IKKα/β were measured by Western blotting assay. The results indicated that knockdown of TFF1 significantly promoted the phosphorylation of NF-κB pathway-associated proteins (*P*<0.05, Fig. 4b). Flow cytometry was used to detect the apoptosis rate of ARPE-19 cells. The data indicated that knockdown of TFF1 significantly promoted HG-induced apoptosis (*P*<0.05). However, BAY 11–7082, an inhibitor of NF-κB pathway, restored the apoptosis rate induced by TFF1 knockdown (*P*<0.05, Fig. 4c). The Western blotting data suggested that knockdown of TFF1 significantly increased the expression of cleaved-caspase 3 and Bax and inhibited Bcl-2 expression, while BAY 11–7082 treatment reversed this phenomenon (*P*<0.05, Fig. 4d). Therefore, the above results reveal that the knockdown of TFF1 promotes HG-induced apoptosis in RPE cells by activating the NF-κB pathway.

### NFE2 regulates TFF1-mediated NF-κB signalling by upregulating miR-423-5p in ARPE-19 cells

**Fig. 5:**
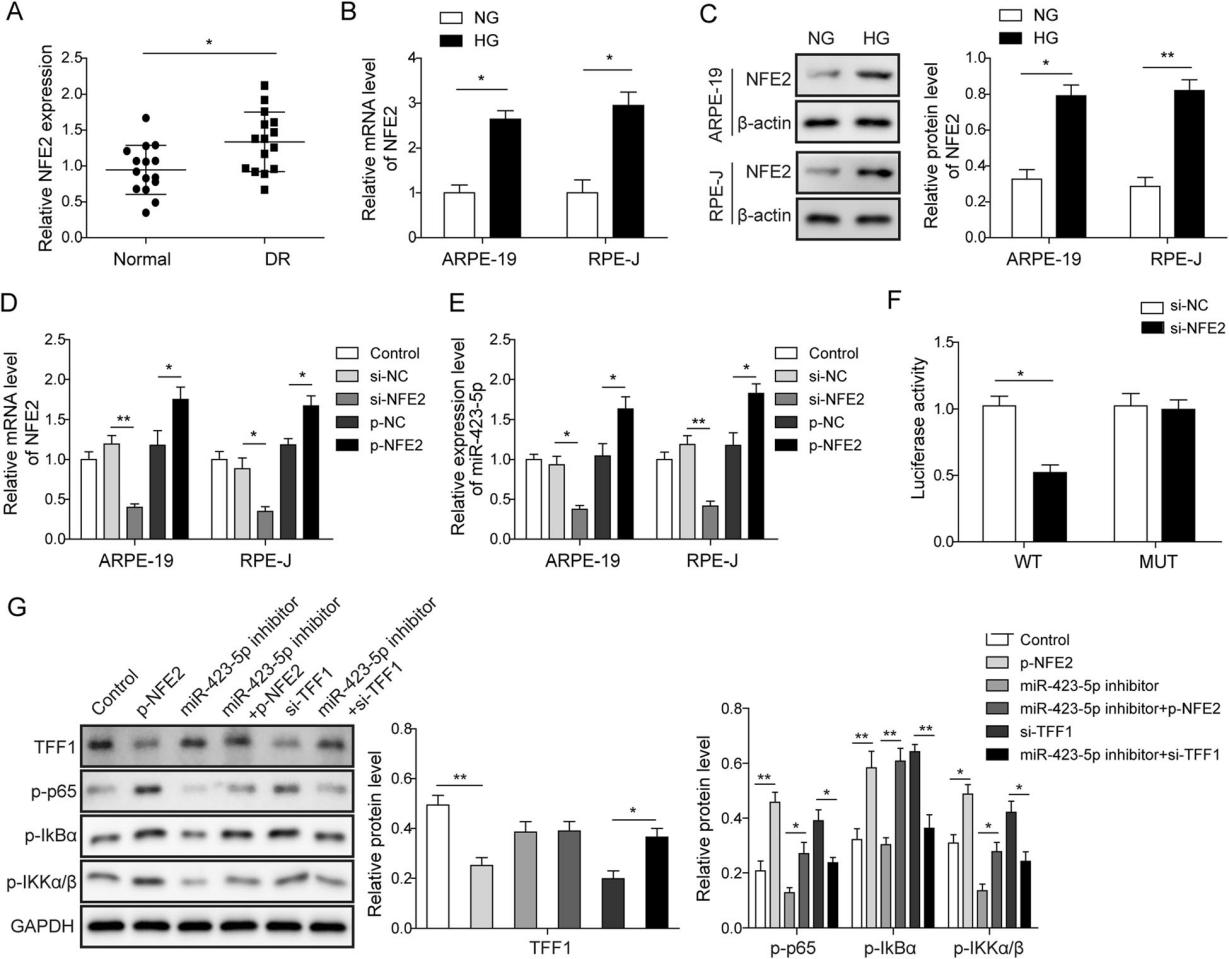
NFE2 regulates the TFF1-mediated NF-κB pathway by upregulating miR-423-5p in ARPE-19 cells. **a** The relative levels of NFE2 were measured by qRT-PCR, which showed that NFE2 was upregulated in DR group. For (**b**) and (**c**), ARPE-19 and RPE-J cells were treated with NG or HG. The relative mRNA (**b**) and protein levels (**c**) of NFE2 were measured by qRT-PCR and Western blot, respectively. The data showed that expression of NFE2 was significantly increased in the HG group. For (**d**) and (**e**), ARPE-19 and RPE-J cells were divided into five groups: control group, si-NC group, si-NEF2 group, p-NC group and p-NEF2 group. **d** Results from qRT-PCR manifested that si-NEF2 or p-NEF2 was successfully transfected. **e** miR-423-5p expression were measured by qRT-PCR, which showed that knockdown of NFE2 significantly inhibited miR-423-5p expression, whereas the overexpression of NFE2 significantly promoted miR-423-5p expression. **f** A dual-luciferase reporter experiment was performed to assess the luciferase activity of reporters in the indicated cells. The relative luciferase activity is the ratio of the luciferase activity in each test cell to that in control cells. **g** The relative protein levels of TFF1, p-p65, p-IκBα and p-IKKα/β were assessed by Western blot. The results showed that overexpression of NFE2 inhibited the expression of TFF1, and knockdown of miR-423-5p increased the expression of TFF1. The knockdown of TFF1 activated the NF-κB pathway, and the knockdown of miR-423-5p inhibited the NF-κB pathway via TFF1. Overexpressed of NFE2 activates the NF-κB pathway through the miR-423-5p-mediated regulation of TFF1. Data are presented as mean ± SD. Three replicate wells were used per sample in each experiment and experiments were replicated three times. * *P* < 0.05; ** *P* < 0.01

According to previous reports, the transcription factor NFE2 directly regulates miR-423-5p expression [10]. First, qRT-PCR was conducted to detect the expression of NFE2 in plasma samples, and we found that NFE2 was significantly upregulated in the plasma of DR patients (Fig. 5a). The regulatory relationship between NFE2 and miR-423-5p was further verified in ARPE-19 cells. The results of qRT-PCR and Western blotting assays are shown in Fig. 5b-c. Compared with the NG group cells, both the mRNA and protein levels of NFE2 were significantly increased in the HG group ARPE-19 and RPE-J cells. Subsequently, knockdown and overexpression of NFE2 was performed by transfection of si-NFE2 and p-NFE2, respectively. The transfection of si-NFE2 resulted in a significant decrease in NFE2 expression, while transfection of p-NFE2 resulted in a significant increase in the mRNA levels of NFE2 (*P*<0.05, Fig. 5d). Furthermore, the knockdown of NFE2 significantly inhibited miR-423-5p expression, whereas the overexpression of NFE2 significantly promoted miR-423-5p expression (*P*<0.05, Fig. 5e). To further determine whether NFE2 regulates miR-423-5p promoter activity in RPE cells, ARPE-19 cells were subjected to the dual-luciferase reporter assay. The cells were transiently transfected with si-NEF2 or the corresponding control. Then, these cells were co-transfected with the miR-423-5p promoter-luciferase reporter construct pGL3-miR-423-5p (containing the NFE2-binding element) or pGL3-miR-423-5p-MUT (lacking the NFE2-binding element). The results demonstrated that the reporter activity of pGL3-miR-423-5p was significantly decreased in the miR-423-5p-overexpressing ARPE-19 cells compared to the corresponding control cells (*P*<0.05, Fig. 5f). In contrast, the reporter activity of pGL3-miR-423-5p-MUT was not significantly changed by NEF2 overexpression. Finally, Western blotting assays were performed with ARPE-19 cells to detect the expression of TFF1 and NF-κB pathway-related proteins p-p65, p-IκBα, and p-IKKα/β. The results showed that overexpression of NFE2 inhibited the expression of TFF1, and knockdown of miR-423-5p increased the expression of TFF1. The knockdown of TFF1 activated the NF-κB pathway, and the knockdown of miR-423-5p inhibited the NF-κB pathway via TFF1. Overexpressed of NFE2 activates the NF-κB pathway through the miR-423-5p-mediated regulation of TFF1 (Fig. 5g). Overall, NFE2 increases the expression of miR-423-5p and further activates the NF-κB pathway by inhibiting TFF1 in HG-induced RPE cells.

## Discussion

Hyperglycaemia is generally considered to be an important cause of DR pathology [11]. RPE cells are important functional units of the retina. They are arranged neatly to form a single cell layer structure, distributed between the choroid and the retinal neuroepithelial layer. RPE cells participate in the process of maintaining the blood-retinal barrier, the expression and secretion of growth-promoting factors, the regulation of intraocular ion balance, and the improvement of visual cycle metabolism [10]. However, under the influence of pathological factors and exogenous stimulating factors, for instance, high glucose, the damage to RPE cells can trigger the dysregulation of intracellular metabolism, leading to the destruction of the retinal environment and the occurrence of retinopathy damage [12]. In this study, we established an HG-induced RPE cell model to mimic the pre-reaction of human hyperglycaemia on retinal function.

It has been reported that miR-423-5p expression is upregulated in PDR eyes [9]. Hence, we suspected that miR-423-5p might be associated with DR progression. Consistent with a previous study, our study suggests that the expression of miR-423-5p is increased in DR patient plasma and HG-induced RPE cells, indicating that miR-423-5p play a potential role in DR. Most recent studies have also indicated that miR-423-5p is involved in regulating cell proliferation and apoptosis [13–15]. Recent research has shown that miR-423-5p inhibits myoblast proliferation and differentiation by targeting Sufu [15]. Another study showed that miRNA-423-5p facilitates hypoxia/reoxygenation-induced apoptosis in renal proximal tubular epithelial cells [14]. Consistent with these findings, our present work demonstrates that miR-423-5p promotes HG-induced apoptosis in RPE cells. MiR-423-5p actually plays a role in promoting HG-included RPE apoptosis.

Furthermore, our results indicate that TFF1 is downregulated in DR patient plasma and HG-induced RPE cells, indicating that TFF1 might be involved in DR progression. TFF1, a group of small molecule polypeptides, which is a member of the trefoil factor family, is involved in various physiological processes, such as central nervous system regulation, gastrointestinal smooth muscle peristalsis, mucosal protection and wound repair [16–19]. Additionally, TFF1 has been found to inhibit the occurrence and development of tumours as a tumour suppressor [20, 21]. In recent years, it has been found that TFF1 function in cell migration, proliferation, differentiation, and blood vessel formation [19, 21]. Additionally, TFF1 can delay the transformation of cells from Gl phase to S phase, thereby reducing apoptosis [22]. Consequently, we hypothesized that TFF1 might play central roles in the regulation of HG-included RPE apoptosis. Subsequent functional experiments in our study demonstrated that miR-423-5p directly targets TFF1 and inhibits TFF1 expression, and TFF1 overexpression inhibits apoptosis in HG-treated RPE cells. Moreover, the upregulation of TFF1 abrogates the miR-423-5p-mediated pro-apoptosis effects in HG-induced RPE cells. Hence, we conclude that miR-423-5p exerts its pro-apoptotic effect in HG-induced RPE cells by suppressing TFF1.

In addition, a study in gastric cancer showed that the loss of TFF1 is associated with the activation of NF-κB [23]. Nuclear factor NF-κB is a transcription factor with multiple regulatory functions in cells and regulates cell proliferation, differentiation, and apoptosis [24]. Studies have shown that the activity of NF-κB may be associated with DR [25, 26]. NF-κB activation is upregulated in non-proliferative diabetic retinopathy (NPDR) and PDR [27]. Increased O-GlcNAcylation of NF-κB enhances retinal ganglion cell death in streptozotocin-induced DR [26]. In this study, knockdown of TFF1 activated the NF-κB pathway and promoted HG-induced apoptosis in RPE cells. Therefore, we can conclude that miR-423-5p regulates HG-induced apoptosis in RPE cells by inhibiting TFF1-mediated NF-κB signalling.

We previously studied the function and regulation mechanism of miR-423-5p in promoting apoptosis in high glucose-cultured RPE cells. However, the upstream regulatory mechanism of miR-423-5p was not clear. The transcriptional factor NFE2 belongs to the Cap’n’Collar basic leucine zipper (CNC-bZIP) family, which includes NFE1, NFE2, and LCR-F1 [28]. A previous study showed that the transcription factor NFE2 binds to the promoter of the miR-423-5p precursor and positively regulates miR-423-5p expression in type 2 diabetes [28]. Studies have shown that NFE2 improves hepatic insulin sensitivity and whole-body insulin resistance [29]. Although NFE2 has been demonstrated to be involved in the regulation of glucose metabolism, the role of NFE2 in DR remains unknown. In the present study, we verified that the expression of NFE2 was increased in DR patient plasma as well as in HG-induced RPE cells. Furthermore, our findings revealed that NFE2 facilitates miR-423-5p expression in HG-induced RPE cells. Mechanistically, NFE2 regulates TFF1-mediated NF-κB signalling by up-regulating miR-423-5p in RPE cells.

## Conclusions

In conclusion, we identified a novel signalling axis, NFE2-miR-423-5p-TFF1-NF-κB. Given the important roles of this signalling axis in regulating HG-induced RPE apoptosis, this novel finding suggests a new direction for the study and treatment strategies of DR, for instance, the development of inhibitors targeting NFE2, miR-423-5p, or activators targeting TFF1, and exploring whether the target has a synergistic (or anti-antagonistic) effect with the DR drugs to reduce (or increase) the drug dose. Our research focused on the cellular level, so we did not establish a DR animal model for related experiments. In future studies, we will further explore the role and mechanism of miR-423-5p in DR at the cell, animal, and clinical levels.

## Methods

### Patient recruitment

This study was approved by the Institutional Ethics Committee of The Second Affiliated Hospital of Zhejiang University School of Medicine, and written informed consent was also obtained from each participant. All 15 patients underwent ophthalmologic examination, including visual acuity, slit lamp examination, fundus contact lens examination and fluorescein angiography. Another 15 volunteers without diabetes mellitus or other malignant diseases were included as the control group. Up to 3 ml of whole blood from each fasting participant was collected, and the samples used in our study were obtained from the blood samples collected in anticoagulant tubes.

### Cell culture

ARPE-19 and RPE-J cells were obtained from the American Type Culture Collection (ATCC, Manassas, VA, USA). Cells were cultured in Dulbecco’s modified Eagle’s medium (DMEM) (Gibco, NY, USA) with 10% foetal bovine serum (FBS) (Gibco, NY, USA) at 37 °C in a 5% CO_2_ atmosphere. The medium was renewed two or three times weekly. The third generation ARPE-19 and RPE-J cell lines in good condition were selected for high glucose (HG) and normal glucose (NG) treatment. According to the previous reference, cells were cultured in 35 mM (HG group) or 5 mM (NG group) D-glucose (Gibco) for 48 h and then examined for subsequent experiments [30, 31].

### Total RNA extraction and quantitative real-time PCR (qRT-PCR) assay

Total RNA was extracted from plasma samples, ARPE-19 and RPE-J cells using TRIzol (Thermo Fisher Scientific, Invitrogen, USA), according to the manufacturer’s protocol. Reverse transcription was performed using a PrimeScript RT reagent kit with gDNA Eraser (Takara, Dalian, China) followed by qRT-PCR using a SYBR Green Realmaster Mix kit (Tiangen, Beijing, China). U6 and GADPH were used as internal standards. The expression levels were calculated according to the 2^−ΔΔCT^ method. The primers used in the PCRs are listed below: NFE2 (sense): 5′-TTCAGCCAGGCTATAAGTCAGG-3′ and NFE2 (antisense): 5′- GCTCAGGATTGGTGGTATGAGA-3′; TFF1 (sense): 5′- CCCTCCCAGTGTGCAAATAAG-3′ and TFF1 (antisense): 5′- GAACGGTGTCGTCGAAACAG-3′; GAPDH (sense): 5′-TGTGGGCATCAATGGATTTGG-3′ and GAPDH (antisense): 5′-ACACCATGTATTCCGGGTCAAT-3′. miR-423-5p and U6 expression was assessed using TaqMan miRNA probes (Applied Biosystems, MA, USA). The average of three independent analyses for each gene was calculated.

### Protein extraction and Western blot

Total protein of cells was extracted with Radio-Immunoprecipitation Assay (RIPA) lysis buffer (Pierce, MA, USA) and then separated with 10% sodium dodecyl sulphate–polyacrylamide gel electrophoresis (SDS-PAGE). Subsequently, the protein was transferred to a polyvinylidene difluoride membrane (PVDF, Millipore, MA, USA) and blocked with 5% non-fat milk for 2 h at room temperature. The membranes were incubated with primary antibodies at 4 °C overnight and then incubated with a goat anti-rabbit IgG antibody (1:10000 dilution, #7054, Cell Signaling Technology, MA, USA) at room temperature for 2 h. GADPH and β-actin were used as internal standards. Visualization was performed by using an ECL assay (Millipore). In addition, for the nuclear translocation of p65, nuclear and cytosolic fractions were obtained using NE-PER™ Nuclear and Cytoplasmic Extraction Reagents (Pierce). Western blot analysis was performed as described above. Primary antibodies were as follows: B cell lymphoma-2 (Bcl-2, #4223), Bcl-2 associated X protein (Bax, #5023), cleaved-caspase 3 (#9664), TFF1 (#15571), phospho-NF-κB p65 (Ser536) (p-p65, #3033), phospho-IκBα (Ser32/Ser36) (p-IκBα, #2859), phospho-IKKα/β (Ser176/180) (p-IKKα/β, #2697), GAPDH (#5174) and β-actin (#4970). All of the primary antibodies were purchased from Cell Signaling Technology and used at a dilution of 1:1000.

### Plasmid construction and cell transfection

The pcDNA3.1-NEF2 (p-NEF2) and pcDNA3.1-TFF1 (p-TFF1) plasmids were obtained from Addgene (Cambridge, MA, USA). The targeting sequences were synthesized by GenePharma Co., Ltd. (Shanghai, China). Small interfering RNA (siRNA) sequences targeting TFF1 or NFE2 were as follows: siRNA-NC (si-NC; UUCUCCGAACGUGUCACGUTT), siRNA-TFF1 (si-TFF1; 5′- ACAAUUCUGUCUUUCACGGGG-3′) and siRNA-NFE2 (si-NFE2; 5′- AAGAUGAUGACAGUAUAUGCU-3′). The sequences for the overexpression or silencing of miR-423-5p were as follows: miR-423-5p mimic (5′-UGAGGGGCAGAGAGCGAGACUUU-3′), negative control (NC) mimic (5′-UCACAACCUCCUAGAAAGAGUAGA-3′), miR-423-5p inhibitor (5′-UGAGGGGCAGAGAGCGAGACUUU-3′), NC inhibitor (5′-UCACAACCUCCUAGAAAGAGUAGA-3′). ARPE-19 and/or RPE-J cells were seeded in 6-well plates and incubated for 18 h before transfection. Plasmids were transfected into cells using Lipofectamine 2000 (Invitrogen, USA) according to the manufacturer’s protocol. The transfection efficiency was determined by fluorescence microscopy using fluorescein-labelled genes, and the expression level was evaluated by qRT-PCR. Transfected cells were collected after 48 h for analysis.

### Flow cytometry assay

Apoptosis was evaluated by flow cytometry using an Annexin V-FITC and propidium iodide (PI) kit (KeyGen Biotech, Nanjing, China). Briefly, ARPE-19 and RPE-J cells were seeded in 6-well plates (1 × 10^6^ cells per well) and then incubated at 37 °C in 5% CO_2_ after transfection for 48 h. Subsequently, the cells were harvested and washed 3 times with PBS and then stained with PI-conjugated anti-Annexin V antibodies under darkness for 20 min at room temperature. Finally, flow cytometry (Becton, New Jersey, USA) was performed to assess the apoptosis rate.

### Dual-luciferase reporter assay

StarBase v2.0 (http://starbase.sysu.edu.cn/index.php) and microRNA.org (http://www.microrna.org) were used for the bioinformatic analysis to predict the binding sites of TFF1 or NFE2 targeted by miR-423-5p. Cells were cultured and inoculated in 24-well plates, followed by transfection. The 3′-UTR sequence of TFF1 or NFE2 containing the presumed miR-423-5p binding sites (WT) or mutated binding sites (MUT) were inserted into the pGL3 (Promega, WI, USA) plasmid. Putative miR-423-5p binding sites or mutated binding sites and the NC mimic, miR-423-5p mimic, NC inhibitor or miR-423-5p inhibitor were co-transfected into ARPE-19 cells by Lipofectamine 2000 (Invitrogen). In addition, fragments of the miR-423-5p promoter region with or without NFE2 binding sites were cloned into the luciferase reporter gene plasmid pGL3. The pGL3 reporter and si-NFE2 were transiently co-transfected into ARPE-19 cells. Luciferase activity after transfection for 48 h was measured by a Dual-Luciferase Reporter Assay kit (Promega).

### Statistical analysis

Three replicate wells were used per sample in each experiment and experiments were replicated three times. SPSS 22.0 statistical software (Version 22.0 SPSS, Chicago, IL, USA) was performed for statistical data analysis. Data are presented as the mean ± standard deviation (mean ± SD). Student’s *t*-test and one-way ANOVA were applied to determine the statistical significance. *P* < 0.05 was considered to be statistically significant.

## Data Availability

All data generated or analysed during this study are included in this published article [and its supplementary information files].

## Abbreviations

DMEM: Dulbecco’s modified Eagle’s medium
DR: Diabetic retinopathy
FBS: Foetal bovine serum
miRNA: MicroRNA
NC: Negative control
NFE2: Nuclear factor-erythroid 2
NPDR: Non-proliferative diabetic retinopathy
PDR: Proliferative diabetic retinopathy
PI: Propidium iodide
PVDF: Polyvinylidene difluoride
qRT-PCR: Quantitative real-time polymerase chain reaction
RPE: Retinal pigment epithelial
SD: Standard deviation
SDS-PAGE: Sodium dodecyl sulphate–polyacrylamide gel electrophoresis
siRNA: Small interfering RNA
TFF1: Trefoil factor 1

## References

[1] Hendrick AM, Gibson MV, Kulshreshtha A (2015). Diabetic retinopathy. Prim Care.

[2] Gholamhossein Y, Behrouz H, Asghar Z (2014). Diabetic retinopathy risk factors: plasma erythropoietin as a risk factor for proliferative diabetic retinopathy. Korean J Ophthalmol.

[3] Wang Wei, Lo Amy (2018). Diabetic Retinopathy: Pathophysiology and Treatments. International Journal of Molecular Sciences.

[4] Gebert LFR, MacRae IJ (2019). Regulation of microRNA function in animals. Nat Rev Mol Cell Biol.

[5] Liu Q (2018). Capturing intracellular oncogenic microRNAs with self-assembled DNA nanostructures for microRNA-based cancer therapy. Chem Sci.

[6] Mastropasqua R (2014). Role of microRNAs in the modulation of diabetic retinopathy. Prog Retin Eye Res.

[7] Qing S (2014). Serum miRNA biomarkers serve as a fingerprint for proliferative diabetic retinopathy. Cell Physiol Biochem.

[8] Kovacs B (2011). MicroRNAs in early diabetic retinopathy in streptozotocin-induced diabetic rats. Invest Ophthalmol Vis Sci.

[9] Hirota K (2015). Comparisons of microRNA expression profiles in vitreous humor between eyes with macular hole and eyes with proliferative diabetic retinopathy. Graefes Arch Clin Exp Ophthalmol.

[10] Zhou W (2011). The role of SLIT-ROBO signaling in proliferative diabetic retinopathy and retinal pigment epithelial cells. Mol Vis.

[11] Nyengaard JR (2004). Interactions between hyperglycemia and hypoxia: implications for diabetic retinopathy. Diabetes.

[12] Chen YH (2012). Effect of high glucose on secreted proteome in cultured retinal pigmented epithelium cells: its possible relevance to clinical diabetic retinopathy. J Proteome.

[13] Liu J (2014). miRNA423-5p regulates cell proliferation and invasion by targeting trefoil factor 1 in gastric cancer cells. Cancer Lett.

[14] Yuan XP (2017). MicroRNA-423-5p facilitates hypoxia/reoxygenation-induced apoptosis in renal proximal tubular epithelial cells by targeting GSTM1 via endoplasmic reticulum stress. Oncotarget.

[15] Ge J (2018). miR-423-5p inhibits myoblast proliferation and differentiation by targeting Sufu. J Cell Biochem.

[16] Yamaguchi J (2015). Pancreatic duct glands (PDGs) are a progenitor compartment responsible for pancreatic ductal epithelial repair. Stem Cell Res.

[17] Perry JK (2008). Are trefoil factors oncogenic?. Trends Endocrinol Metab.

[18] Jensen P (2015). Effects of Forskolin on trefoil factor 1 expression in cultured ventral mesencephalic dopaminergic neurons. Neuroscience.

[19] Mathelin C, Tomasetto C, Rio MC (2005). Trefoil factor 1 (pS2/TFF1), a peptide with numerous functions. Bull Cancer.

[20] Aihara E, Engevik KA, Montrose MH (2017). Trefoil factor peptides and gastrointestinal function. Annu Rev Physiol.

[21] Busch M (2017). Reduction of the tumorigenic potential of human retinoblastoma cell lines by TFF1 overexpression involves p53/caspase signaling and miR-18a regulation. Int J Cancer.

[22] Bossenmeyer-Pourie C (2002). The trefoil factor 1 participates in gastrointestinal cell differentiation by delaying G1-S phase transition and reducing apoptosis. J Cell Biol.

[23] Soutto M (2011). Loss of TFF1 is associated with activation of NF-kappaB-mediated inflammation and gastric neoplasia in mice and humans. J Clin Invest.

[24] Xia Y, Shen S, Verma IM (2014). NF-kappaB, an active player in human cancers. Cancer Immunol Res.

[25] Mitamura Y (2003). NF-kappaB in epiretinal membranes after human diabetic retinopathy. Diabetologia.

[26] Kim SJ (2016). Increased O-GlcNAcylation of NF-kappaB enhances retinal ganglion cell death in Streptozotocin-induced diabetic retinopathy. Curr Eye Res.

[27] Choudhuri S (2015). Role of NF-kappaB activation and VEGF gene polymorphisms in VEGF up regulation in non-proliferative and proliferative diabetic retinopathy. Mol Cell Biochem.

[28] Yang W (2017). NFE2 induces miR-423-5p to promote gluconeogenesis and hyperglycemia by repressing the hepatic FAM3A-ATP-Akt pathway. Diabetes.

[29] Zhang Y (2018). Exercise induced improvements in insulin sensitivity are concurrent with reduced NFE2/miR-432-5p and increased FAM3A. Life Sci.

[30] Jiang Y, Sang Y, Qiu Q (2017). microRNA-383 mediates high glucose-induced oxidative stress and apoptosis in retinal pigment epithelial cells by repressing peroxiredoxin 3. Am J Transl Res.

[31] Dai C (2019). Baicalin protects human retinal pigment epithelial cell lines against high glucose-induced cell injury by up-regulation of microRNA-145. Exp Mol Pathol.

